# Activation of goblet cell Piezo1 alleviates mucus barrier damage in mice exposed to WAS by inhibiting H3K9me3 modification

**DOI:** 10.1186/s13578-023-00952-5

**Published:** 2023-01-12

**Authors:** Yan Xu, Yilin Xiong, Ying Liu, Gangping Li, Tao Bai, Gen Zheng, Xiaohua Hou, Jun Song

**Affiliations:** Department of Gastroenterology, Union Hospital, Tongji Medical College, Huazhong University of Science and Technology, Wuhan, 430022 China

**Keywords:** Piezo1, H3K9me3, mucin2, Goblet cells, Avoiding water stress

## Abstract

**Background:**

Our recent studies found that intestinal mechanical signals can regulate mucus synthesis and secretion of intestinal goblet cells through piezo type mechanosensitive ion channel component 1 (Piezo1), but the detailed molecular mechanisms remain to be investigated. Previous studies using a water avoidance stress (WAS) model reported decreased intestinal mucus accompanied by abnormal intestinal motility. It has also been reported that the expression of mucin2 was negatively correlated with histone H3 lysine 9 trimethylation (H3K9me3), a key regulator of histone methylation, and that mechanical stimulation can affect methylation. In this study, we aimed to determine whether and how Piezo1 expressed on goblet cells regulates mucus barrier function through methylation modification.

**Methods:**

A murine WAS model was established and treated with Yoda1 (Piezo1 agonist), and specific Piezo1 flox-mucin2 Cre mice were also tested. The mucus layer thickness and mucus secretion rate of mouse colonic mucosa were detected by a homemade horizontal Ussing chamber, intestinal peristaltic contraction was detected by the ink propulsion test and organ bath, goblet cells and mucus layer morphology were assessed by HE and Alcian blue staining, mucus permeability was detected by FISH, and the expression levels of Piezo1, H3K9me3 and related molecules were measured by Western blots and immunofluorescence. LS174T cells were cultured on a shaker board in vitro to simulate mechanical stimulation. Piezo1 and H3K9me3 were inhibited, and changes in mucin2 and methylation-related pathways were detected by ELISAs and Western blots. ChIP-PCR assays were used to detect the binding of H3K9me3 and mucin2 promoters under mechanical stimulation.

**Results:**

Compared with those of the controls, the mucus layer thickness and mucus secretion rate of the mice exposed to WAS were significantly decreased, the mucus permeability increased, the number of goblet cells decreased, and the intestinal contraction and peristalsis were also downregulated and disordered. Intraperitoneal injection of Yoda1 improved mucus barrier function and intestinal contraction. In the colonic mucosa of mice exposed to WAS, Piezo1 was decreased, and histone H3 lysine 9 trimethylation (H3K9me3) and methyltransferase suppressor of variegation 3–9 homolog 1 (SUV39h1) were increased, but activating Piezo1 alleviated these effects of WAS. Piezo1 flox-mucin2 Cre mice showed decreased mucus expression and increased methylation compared to wild-type mice. Cell experiments showed that mechanical stimulation induced the activation of Piezo1, decreased H3K9me3 and SUV39h1, and upregulated mucin2 expression. Inhibition of Piezo1 or H3K9me3 blocked the promoting effect of mechanical stimulation on LS174T mucin2 expression. The binding of H3K9me3 to the mucin2 promoter decreased significantly under mechanical stimulation, but this could be blocked by the Piezo1 inhibitor GsMTx4.

**Conclusion:**

Piezo1 mediates mechanical stimulation to inhibit SUV39h1, thereby reducing H3K9me3 production and its binding to the mucin2 promoter, ultimately promoting mucin2 expression in goblet cells. This study further confirmed that piezo1 on goblet cells could regulate mucus barrier function through methylation.

**Supplementary Information:**

The online version contains supplementary material available at 10.1186/s13578-023-00952-5.

## Introduction

Mucin2 is the core component of the mucus barrier of the colon and is mainly synthesized and secreted by intestinal goblet cells. Previous research has suggested that this process is regulated by various factors, such as neurotransmitters and immune signals [1]. Our previous research showed for the first time that mechanical stimulation at a certain intensity in the intestine can act on Piezo1 to directly promote mucin2 expression in goblet cells [2]. However, due to the complex downstream pathways of Piezo1 [3], the specific cellular and molecular pathways regulating mucin2 synthesis remain to be further clarified.

Epigenetic modification has been widely recognized in the dynamic regulation of genes, and it plays an important role in the regulation of many physiological processes, including respiratory mucus expression [4, 5]. Histone methylation induced by methyltransferases, such as SUV39h1, is an important form of epigenetic modification that can silence the expression of target genes [6]. Epigenetic modification is closely related to the expression of mucin2 in epithelial cells, methylation of the mucin2 promoter can inhibit its activation [7], and mucin2 hypomethylation is associated with high expression of mucin2 in colon cancer cells [8, 9]. In addition, we have proven that water avoidance stress increased H3K9 methylation, leading to downregulation of occludin expression and an increase in paracellular permeability. Intrarectal administration of an H3K9 methylation antagonist prevented chronic stress-induced visceral hyperalgesia in rats [10]. This suggests that methylation modification is involved in the pathogenesis of the WAS model. Our previous experiments also found that intestinal mucus thickness decreased in mice exposed to WAS, but it is not clear whether and how epigenetic modification regulates mucus expression in the WAS model.

The decrease in colonic mucus expression in WAS model mice was accompanied by abnormal intestinal motility [11], which resulted in changes in mechanical signals. Mechanical signals in the tissue microenvironment are involved in the epigenetic modification of histones [12, 13] to adjust cellular function over time, in which the mechanical transduction pathway plays a key role. Piezo1, a mechanically sensitive ion channel on intestinal goblet cells that can sense intestinal mechanical signals [2], may be the link between abnormal mechanical signals in the intestinal tract of mice exposed to WAS and the methylation of goblet cells.

In this study, Piezo1 function was studied in WAS model mice, and LS174T cells were treated with mechanical stimulation to simulate colonic goblet cells. The expression of mucin2 and the methylation pathways were detected. We found that the colonic mucus barrier function of WAS model mice was impaired, accompanied by a decrease in intestinal motility, downregulation of Piezo1 expression and enhancement of H3K9me3 modification. We also confirmed that Yoda1 and mechanical signals activated Piezo1 to downregulate SUV39h1 expression, thus reducing H3K9me3 expression, decreasing its binding to the mucin2 promoter and finally promoting mucin2 synthesis.

## Methods

### Study design

#### Animals and water avoidance stress (WAS)

Animals were raised in the Animal Experimental Center of Tongji Medical College. As in previous research [14, 15], 9-week-old male C57BL/6J mice were placed on a small platform surrounded by water at a temperature of 25 °C for 2 h per day for 10 days. Mice were randomly divided into three groups (n = 10): the control group, WAS group and WAS + Yoda1 group. In the WAS + Yoda1 group, 1 h before being subjected to WAS, the mice were injected intraperitoneally with Yoda1 (50 μmol/L, 4 ml kg^−1^ day^−1^; Tocris); the mice were treated in this manner consecutively for 10 days.

Mice carrying a floxed Piezo1 locus (Piezo1^LoxP/LoxP^) on a C57BL/6 J background were crossed with mice expressing a tamoxifen-inducible Cre-recombinase transgene under the control of a Mucin2 promoter (Mucin2-CreER^T2^) to generate Piezo1^LoxP/WT^; Mucin2-CreER^T2^ mice (with the capacity to induce a heterozygous deletion of intestinal Piezo1 after tamoxifen treatment). Subsequent cross-breeding of Piezo1^LoxP/WT^; Mucin2-CreER^T2^ mice resulted in the generation of Piezo1 flox-mucin2 Cre mice (Piezo1 flox-mucin2 Cre mice, with the capacity to induce a homozygous deletion of intestinal Piezo1 after tamoxifen treatment). Cre was induced in 8–12-week-old male mice by intraperitoneal injection of tamoxifen (1 mg/day in sunflower oil for 5 days; Sigma, Millipore, St. Louis), while tamoxifen-treated Piezo1^LoxP/LoxP^ mice were used as controls.

### Abdominal withdrawal reflex (AWR)

Visceral sensitivity to colorectal distention (CRD) was measured by the AWR test as described previously [16]. CRD was conducted at constant pressures of 20-, 40-, 60- and 80-mm Hg by a custom-made distension control device. The responses were considered stable if there was less than 20% variability between 2 consecutive trials of CRD at 60 mm Hg. The AWR score was recorded as described in previous research [17].

### Horizontal Ussing chamber and mucus thickness measurement

According to previous research [18], we made a horizontal perfusion chamber. For the ex vivo experiments, mice were anesthetized with isoflurane (R510-22, RWD Life Science Co., Ltd) and killed by cervical dislocation. The distal colon was dissected and flushed, and the muscle layer was removed. The mucosal tissue explant was mounted in a horizontal perfusion chamber. The upper surface of the colonic mucus was visualized by the addition of charcoal particles. The mucus thickness was determined by measuring the distance between the epithelial surface and the mucus surface by a micropipette attached to a patch clamp viewed through a stereomicroscope. Specifically, the height of the micropipette attached to the patch clamp was noted on the stereoscopic microscope using the homemade spatial positioning device. When the front end of the micropipette is located on the mucus surface, the corresponding height is recorded as H1. While keeping the horizontal position unchanged, when the front end of the descending micropipette falls into contact with the epithelial surface, the corresponding height is recorded as H2. H1 minus H2 to get the mucus thickness of the corresponding position. The mean value was calculated after repeated measurement for 3 times, then the position of the micropipette was changed and the mucus thickness at 6 different positions was measured.

### Colonic smooth muscle contraction

Colonic smooth muscle contraction was recorded as previously described [19]. The mean contractile amplitude of each strip was recorded with an RM6240 multichannel physiological signal system. A 1-cm segment of the distal colon longitudinal muscle strip was removed and placed in an organ bath system containing oxygenated (95% O_2_ + 5% CO_2_) Krebs solution (119 mmol/L NaCl, 4.7 mmol/L KCl, 25 mmol/L NaHCO_3_, 1.2 mmol/L NaH_2_PO_4_, 2.5 mmol/L CaCl_2_, 1.2 mmol/L MgSO_4_, and 11.1 mmol/L glucose; pH 7.30–7.40) at 37 °C. The mean contractile amplitude of each strip was recorded with a LabChartReader_8.1.13 multichannel physiological signal system.

Spontaneous smooth muscle activity was recorded, and the motility index (MI) was calculated. The MI is defined as the area under the contractile curve in unit time, which could be considered a comprehensive evaluation of muscle activity containing muscle tension, amplitude, and frequency. Additionally, 10 mM acetylcholine (Ach; A6500, Sigma, Millipore, St. Louis) was added into the bath chamber to observe the excitatory nerve–mediated contractions.

### Ink propulsion test

For measurement of the intestinal transit rate (ITR), mice received an oral administration of 200 μL of Indian ink. The specific process was conducted as described in previous literature [20]. The percentage of blackened intestinal tracts was calculated: ITR (%) = pushing length/total length × 100%.

### Hematoxylin and eosin (H&E) and Alcian blue staining

After the mice were sacrificed, the mouse colon samples were fixed in 10% neutral-buffered formalin and Carnoy’s solution (60% methanol, 30% chloroform, 10% glacial acetic acid, G1120, Servicebio) overnight and then processed by standard methods and embedded in paraffin. H&E and Alcian blue were used to stain paraffin section (3–5 μm) for mucus characterization, which was performed according to a standard protocol [21]. The investigators photographed the sections by using a BX51 Olympus microscope. Another two investigators who were not clear about the grouping measured the Mucus layer thickness (5 measurements per section/2 sections per animal/5 animals per group, 200X) and goblet cells/epithelial cells (10 measurements per section/2 sections per animal/5 animals per group, 200X) with ImageJ software respectively [22].

### Fluorescence in situ hybridization (FISH)

As in previous research [23], paraffin-embedded Carnoy’s-fixed sections were hybridized with 10 ng/ml of a general bacterial 16S rRNA probe (EUB338, red, 5′-GCT GCC TCC CGT AGG AGT-3′, Boster) and immunostained for Mucin2 using the UEA-1 (Bauhinia bean lectin, FL-1061-2, Vector labs). Images were observed with a Hitachi U8010 transmission electron microscope (Hitachi, Ltd., Tokyo, Japan).

### Western blot (WB) and immunofluorescence

Immunoblotting was conducted as described previously [24]. Colon tissues from mice were dissected the next day after completing the WAS procedure. The epithelial layers were gently scraped off, and proteins were extracted. In separate studies, human LS174T cells were collected and processed with the same method. Proteins were separated and blotted with the following primary antibodies: Piezo1 (1:500, 15939-1-AP, Proteintech), H3K9me3 (1:500, 13969, Cell Signaling Technology), T-H3 (1:500, GTX122148, GeneTex), SUV39h1 (1:500, PA5-29470, Thermo-Fisher), Histone Deacetylase 3 (Hdac3) (1:500, GTX1096679, GeneTex), and GAPDH (1:1000; Proteintech).

For immunofluorescence staining, paraffin sections were stained as described in previous research [25]. Primary antibodies used for incubation were as follows: Piezo1 (1:200, 15939-1-AP, Proteintech), H3K9me3 (1:500, 13969, Cell Signaling Technology), AGR2 (1:200, AF6068, R&D Systems), and Mucin2 (1:200, GTX100664, GeneTex). DAPI (D9542, Sigma, Millipore, St. Louis) was used for nucleic acid staining.

### Cell culture and treatment

Briefly, the LS174T cell line was purchased from Shanghai Zhong Qiao Xin Zhou.

Biotechnology. Cells were cultured in DMEM containing 10% FBS and 1% nonessential amino acids. Culture medium was changed every other day. Piezo1 knockdown (Piezo1-KD) LS174T cells were infected with lentivirus packaging Piezo1 siRNA as described in previous research [2].

For shear force intervention, the cells were inoculated on 24-well and 6-well culture plates (Corning, NY, USA). After growing to fusion, the cells were placed on a rocking board in an incubator. The parameters were 0.3 Hz and shear stress = η × φ × 125 ≈ 0.1 N (η: viscosity of the medium; φ: flow rate) as previous research [26]. The Piezo1 agonist Yoda1, antagonist GsMTx4 (HY-P1410, MCE) and the methylation inhibitor UNC0638 (HY-15273, MCE) were added during mechanical stimulation.

### RT-PCR

Quantitative real-time RT-PCR (RT-PCR) was carried out in a LightCycler 480 system using SYBR Green Transcription Master Mix (Roche Diagnostics, IN, USA) as previously described [27].

Mouse primers were as follows.

GAPDH: forward “5′-TGAAGCAGGCATCTGAGG-3′”; reverse “5′-CGAAGGTGGAAGAGTGGGAG-3′”.

Mucin2: forward “5′-GCTGACGAGTGGTTGGTGAATG-3′”; reverse “5′-GATGAGGTGGCAGACAGGAGAC-3′”.

EDEM1: forward “5′-TGAAAGCATGTGAGGGTAGTG-3′”; reverse “5′-GAGAGAAGGGAAGACAGGATAGA-3′”.

GRP78: forward “5′-AAGAATGAAGGAAAAACAGGACAAAA-3′”; reverse “5′-CAAATGGAGAAGATTCCGCC-3′”.

ATF4: forward “5′-CCACTCCAGAGCATTCCTTTAG-3′”; reverse “5′-CTCCTTTACACATGGAGGGATTAG-3′”.

### ELISA

Samples were obtained according to instructions (Human Mucin 2 ELISA Kit Catalog Number: HM10493, Bioswamp). The concentration of mucin2 in LS174T cells was measured as previously described [2].

### Chromatin immunoprecipitation (ChIP) assay

Chromatin immunoprecipitation was performed with a ChIP kit (26157, Pierce). Specific procedures were conducted as described in previous research [28]. LS174T cells after 24 h of mechanical stimulation and control treatment were evaluated. Chromatin was subjected to immunoprecipitation using the following antibodies: H3K9me3 (1:500, 13,969, Cell Signaling Technology). DNA was finally eluted in elution buffer and used for real-time PCR amplification using the same primer sets as MeDIP-qPCR. Primer: TTGGCATTCAGGCTACAGGG; GGCTGGCAGGGGCG-GTG.

### Statistics

The data were analyzed with SPSS 21.0 and GraphPad Prism 8.0 software. All data in the figures are expressed as the mean ± SEM. Differences between groups were evaluated with either one-way analysis of variance (ANOVA) with the Holm-Sidak post hoc test or with a t test using SigmaPlot. The significance level was set at P value < 0.05 (compared to control *P < 0.05, **P < 0.01 and ***P < 0.001).

## Results

### Impaired mucus barrier function and decreased intestinal motility in WAS model mice

**Fig. 1:**
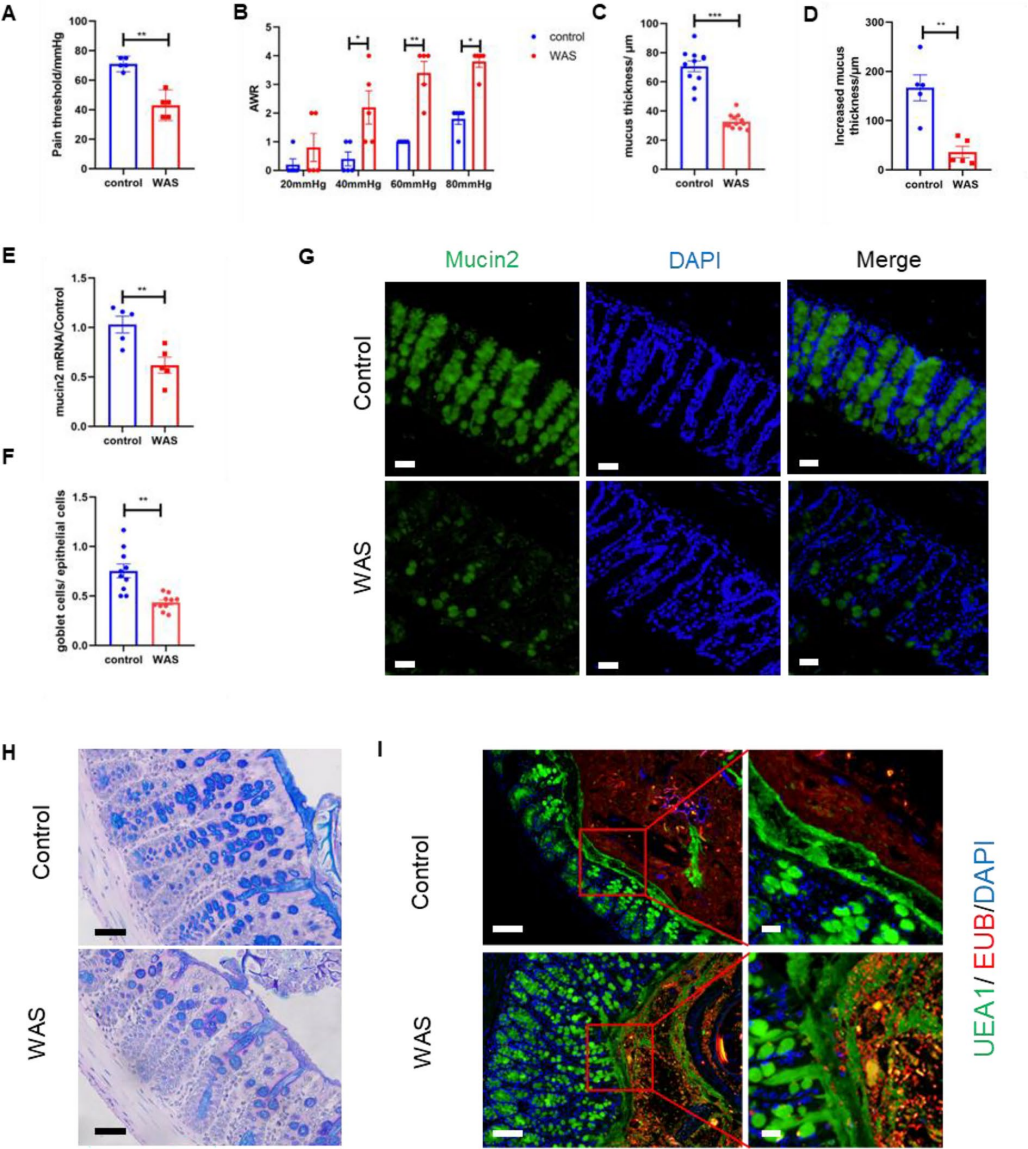
Impaired mucus barrier function in the WAS model mice. **A**, **B** Pain threshold and AWR score in the control and WAS mice (n ≥ 6). **C**, **D** Colon mucus thickness and increased mucus thickness in the control and WAS mice measured by horizontal ussing chamber (n ≥ 6). **E** RT-PCR of mucin2 mRNA in the mucosa of control and WAS mice (n ≥ 6). **F** The ratio of goblet cells to epithelial cells in the control and WAS mice (n ≥ 6). **G** Immunofluorescence of mucin2 in the control and WAS mice; bar length: 50 μm. **H** Alcian staining of control and WAS mice colon; bar length: 50 μm. **I** FISH of control and WAS mice colon, mucus was stained with UEA1 (green), bacteria were detected by fluorescence in situ hybridization with general bacterial 16S probes EUB (red) and DNA stained using DAPI (blue); bar length: 50 μm and 20 μm

The phenomenon of decreased mucus in WAS model rats has been reported [29], but the specific situation in WAS model mice is not clear. We first established a mouse model of WAS to confirm the mucus change. The AWR test was used to verify the success of WAS modeling. The AWR score showed that the WAS group mice responded strongly to colorectal balloon dilatation. The pain threshold of the WAS group was significantly lower than that in the control group (44.3 ± 7.8 mmHg vs. 71.6 ± 3.7 mmHg, P < 0.01), and the AWR score was significantly higher than that in the control group (Fig. 1A, B). Then, we placed the freshly isolated mouse colonic mucosa on a homemade horizontal Ussing chamber to detect the mucus layer thickness. The results showed that the mucus layer thickness of the WAS group was significantly lower than that of the control group (117.4 ± 66.4 μm vs. 267.5 ± 82.6 μm, P < 0.01). After continuous perfusion for half an hour, the increased mucus thickness of the WAS group was lower than that of the control group (39.2 ± 18.8 μm vs. 184.9 ± 33.9 μm, P < 0.05) (Fig. 1C, D), which indicated that the mucus secretion rate of the WAS group decreased. PCR showed that mucin2 mRNA expression decreased in the intestinal mucosa of the mice in the WAS group (Fig. 1E), and the staining results of colonic tissue sections showed that the number of colonic goblet cells and mucus filling decreased in the WAS group (Fig. 1F–H). In addition, FISH showed that some intestinal bacteria penetrated the mucus layer, while the same phenomenon was not observed in the control group, which suggested a slight increase in mucus permeability in the WAS group (Fig. 1I).

**Fig. 2:**
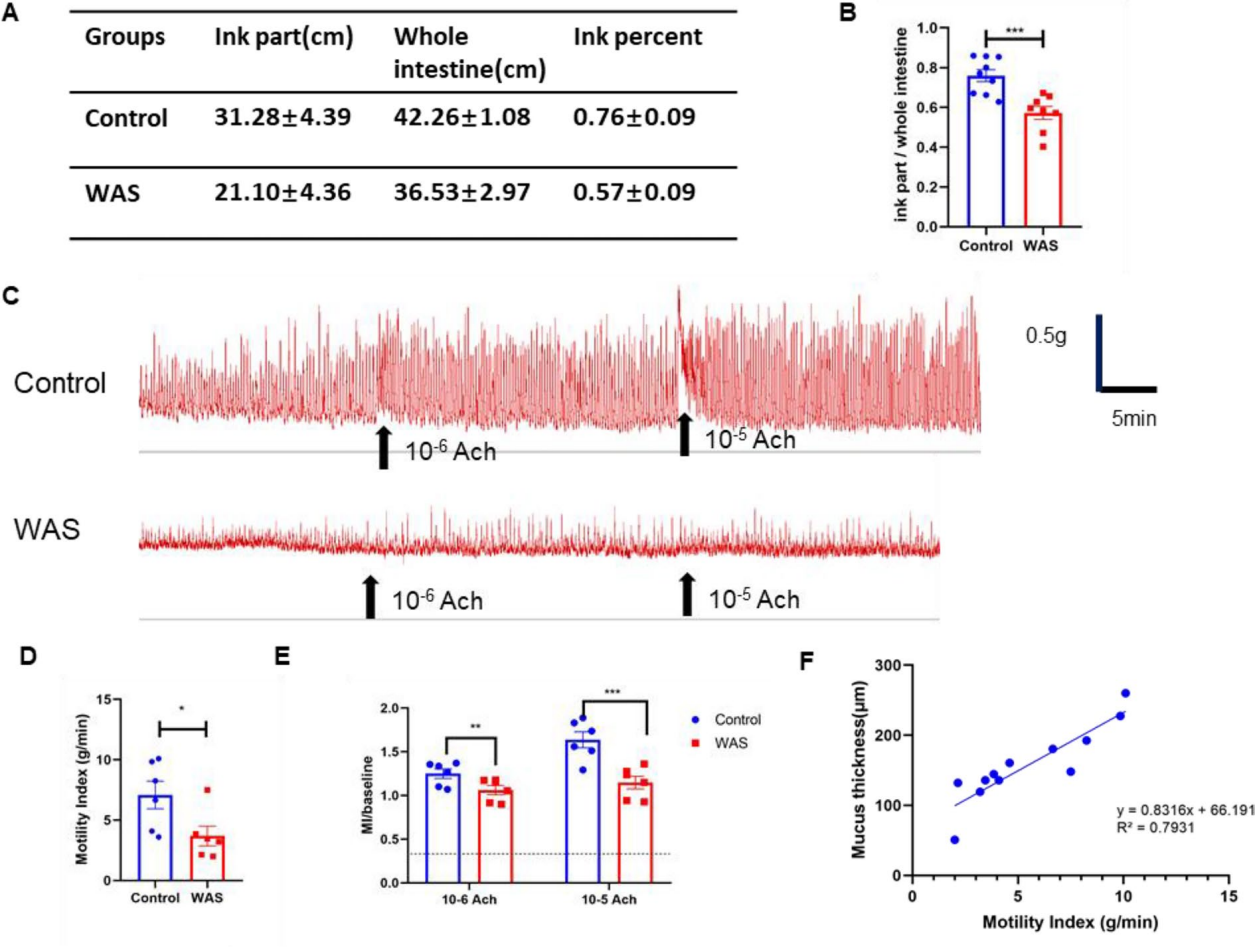
Decreased intestinal motility in the WAS model mice. **A**, **B** Results of the ink propulsion test in the control and WAS groups (n ≥ 5). **C**–**E** Spontaneous activities and acetylcholine (Ach)-induced responses of colonic longitudinal muscles, including MI and MI compared to baseline, in the control and WAS groups (n ≥ 5). **F** Line regression of motility index and mucus thickness of the mice colon

After confirming that intestinal mucus expression was significantly decreased, we focused on another characteristic change in the mice exposed to WAS, that is, intestinal motility dysfunction. We examined intestinal peristalsis and contraction in mice exposed to WAS. The ink propulsion results showed that the ratio of the farthest distance of the ink to the total intestinal length of the mice exposed to WAS was lower than that of the control group (0.57 ± 0.09 vs. 0.76 ± 0.09, P < 0.001), indicating that the intestinal peristaltic propulsion function was decreased significantly (Fig. 2A–B). The organ bath test showed that the mice in the WAS group had a lower MI than the control mice, lost normal contractile rhythm and were insensitive to acetylcholine (Fig. 2C–E). These results showed that the intestinal mechanical signals of WAS model mice were abnormal. In addition, the equation and coefficient of determination obtained by linear regression analysis of the MI and mucus thickness were y = 0.8316x + 66.191 and R^2^ = 0.7931, which indicated a positive correlation between them (Fig. 2F).

### Decreased Piezo1 expression and increased methylation in the WAS model mice

**Fig. 3:**
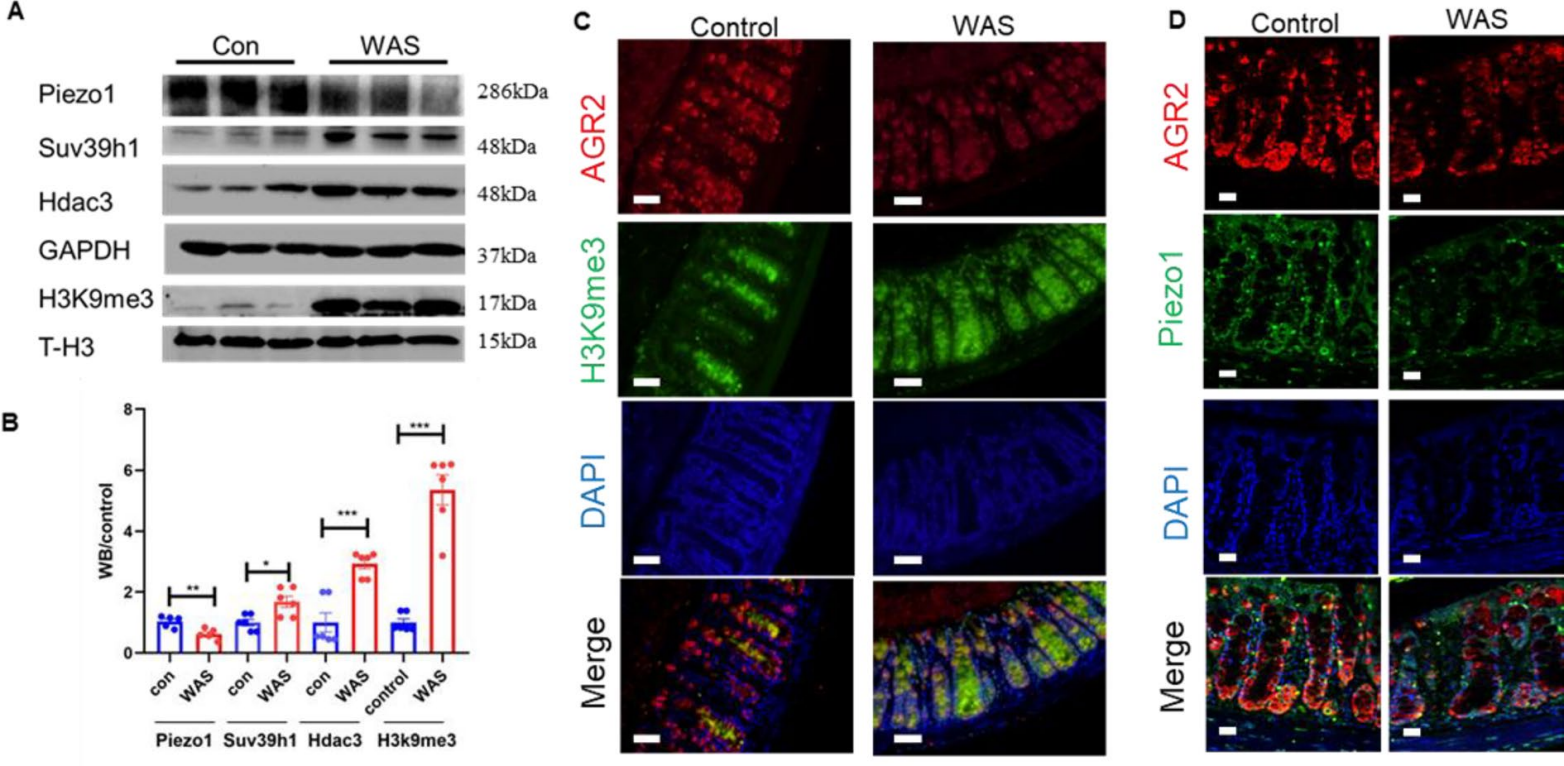
Decreased Piezo1 and enhanced methylation in the intestinal mucosa of WAS model mice. **A** Western blot of Piezo1, Suv39h1, Hdac3, GAPDH, H3K9me3 and T-H3 (Internal parameter for nuclear protein H3K9me3) in the mucosa of control and WAS mice. **B** Statistical analysis of protein expression in panel **A**. **C** Immunofluorescence for co-localization of AGR2 (label of goblet cells) and H3K9me3 in intestinal mucosa of control and WAS mice; bar length: 20 μm. **D** Immunofluorescence for co-localization of AGR2 (label of goblet cells) and Piezo1 in intestinal mucosa of control and WAS mice; bar length: 20 μm

Next, we further explored the Piezo1 and epigenetic changes behind the decrease in intestinal mucus and motility. To explore the epigenetic mechanism of mucus changes in mice exposed to WAS, we specifically isolated the colonic mucosa of the WAS group and control group and detected the level of H3K9 methylation and the expression of related deacetylases and methyltransferases. WB results showed that the expression of H3K9me3 in the colonic mucosa of the WAS group mice was upregulated, and the expression levels of HDAC3 and SUV39h1, which are involved in the regulation of methylation, were also significantly increased (Fig. 3A, B).

To eliminate the interference of other epithelial cells, we used specific antibodies against AGR2 to localize goblet cells and observed the changes in H3K9me3 in goblet cells under a confocal microscope. The immunofluorescence results showed that methylation was increased and the mucin2 level was decreased in goblet cells, which was consistent with the above results (Fig. 3C). In addition, the results of WB and immunofluorescence showed that the expression of Piezo1 in the intestinal mucosa of the mice exposed to WAS decreased (Fig. 3A, B) and that the colocalization of Piezo1 in goblet cells decreased significantly (Fig. 3D).

### The Piezo1 agonist Yoda1 improved mucus barrier function and recovered damaged intestinal motility in the WAS group mice while decreasing methylation

**Fig. 4:**
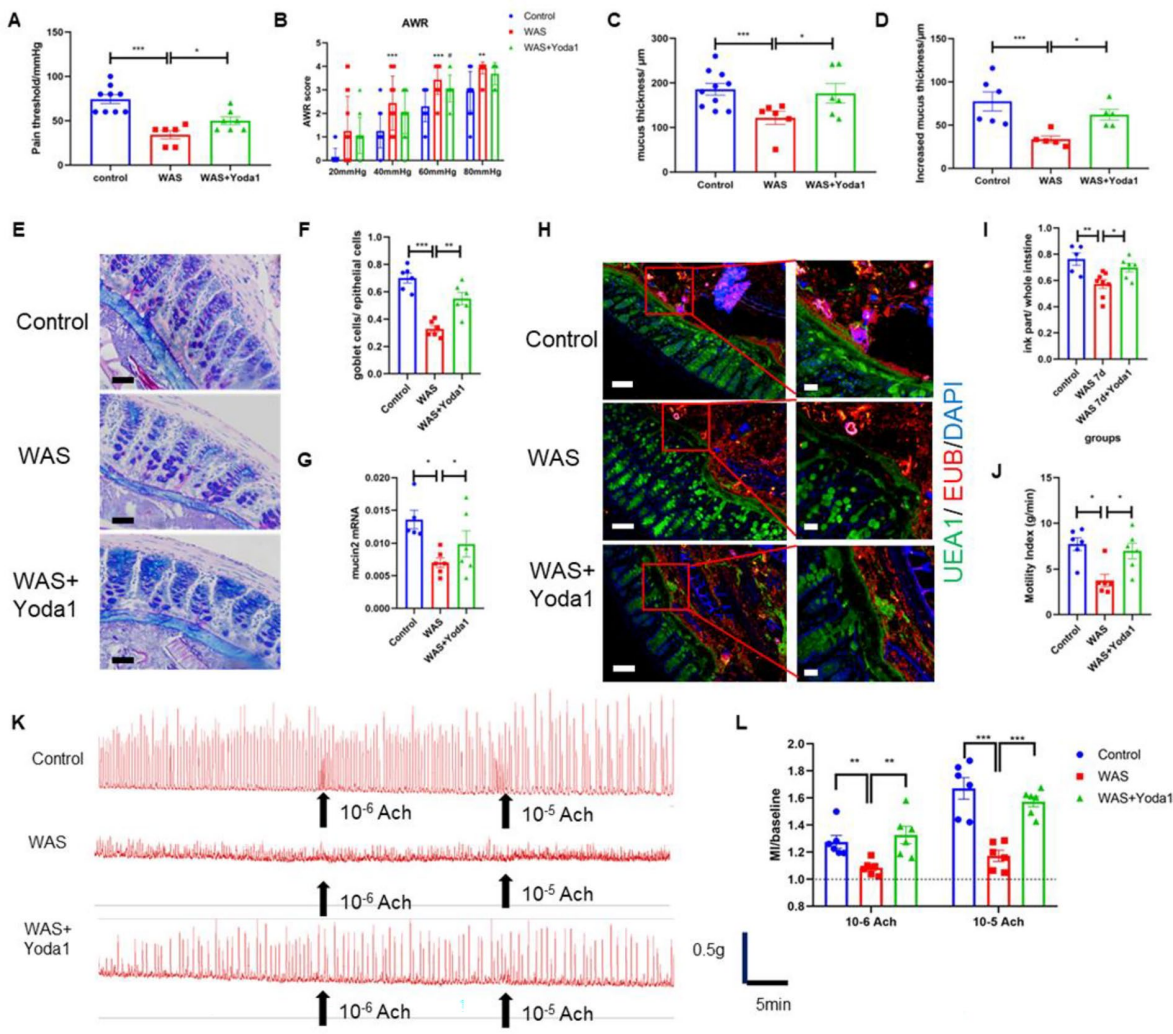
Piezo1 agonist improved mucus barrier function and ameliorated intestinal motility disorder in WAS mice. **A**, **B** Pain threshold and AWR score in the control, WAS and WAS + Yoda1 mice (n ≥ 5). **C**, **D** Colon mucus thickness and increased mucus thickness in the control, WAS and WAS + Yoda1 mice measured by horizontal Ussing chamber (n ≥ 5). **E** Alcian staining of control, WAS and WAS + Yoda1 mice colon; bar length: 50 μm. **F** The ratio of goblet cells to epithelial cells in the control, WAS and WAS + Yoda1 mice (n ≥ 6). **G** RT-PCR of mucin2 mRNA in the mucosa of control, WAS and WAS + Yoda1 mice (n ≥ 5). **H** FISH of control, WAS and WAS + Yoda1 mice colon; mucus was stained with UEA1 (green), bacteria were detected by fluorescence in situ hybridization with general bacterial 16S probes EUB (red) and DNA stained using DAPI (blue); bar length: 50 μm and 20 μm. **I** Results of Ink propulsion test in the control, WAS and WAS + Yoda1 mice. **J**–**L** Spontaneous activities and Acetylcholine (Ach) induced responses of colonic longitudinal muscles, including MI and MI compared to baseline, in the control, WAS and WAS + Yoda1 mice (n ≥ 5)

After confirming that Piezo1 expression was downregulated in the intestinal mucosa of the mice exposed to WAS, we speculated that in vivo intervention with Piezo1 might affect the expression of colonic mucus in these mice. To verify this conjecture, we injected Yoda1, an agonist of Piezo1, into mice by intraperitoneal injection before WAS exposure for 10 days. The results showed that, in Yoda1-treated mice exposed to WAS, the tolerance to colorectal dilatation was slightly increased, the pain threshold was higher (50.0 ± 1.65 mmHg vs. 34.3 ± 1.89 mmHg, P < 0.05), and the mucus thickness (176.8 ± 8.97 μm vs. 121.7 ± 6.03 μm, P < 0.05) and mucus secretion rate (62.3 ± 2.76 μm vs. 34.25 ± 1.67 μm, P < 0.05) were higher (Fig. 4A–F) than those of the WAS group.

Compared to those in the WAS group, the mucus thickness, number of colonic goblet cells and mucin2 mRNA expression in the intestinal mucosa of the Yoda1 group were increased, and FISH showed that the mucus permeability of the Yoda1 group was also improved (Fig. 4G, H). In addition, intestinal propulsion and contraction disorders in the mice exposed to WAS were alleviated after Yoda1 intervention. The motility index of colonic muscle strips of WAS mice treated with Yoda1 was higher than that of the WAS group before the addition of acetylcholine. After the addition of acetylcholine, the increase in the motility index of colonic muscle strips in WAS mice treated with Yoda1 was significantly higher than that in the WAS group and similar to that in the healthy control group (Fig. 4I–L).

**Fig. 5:**
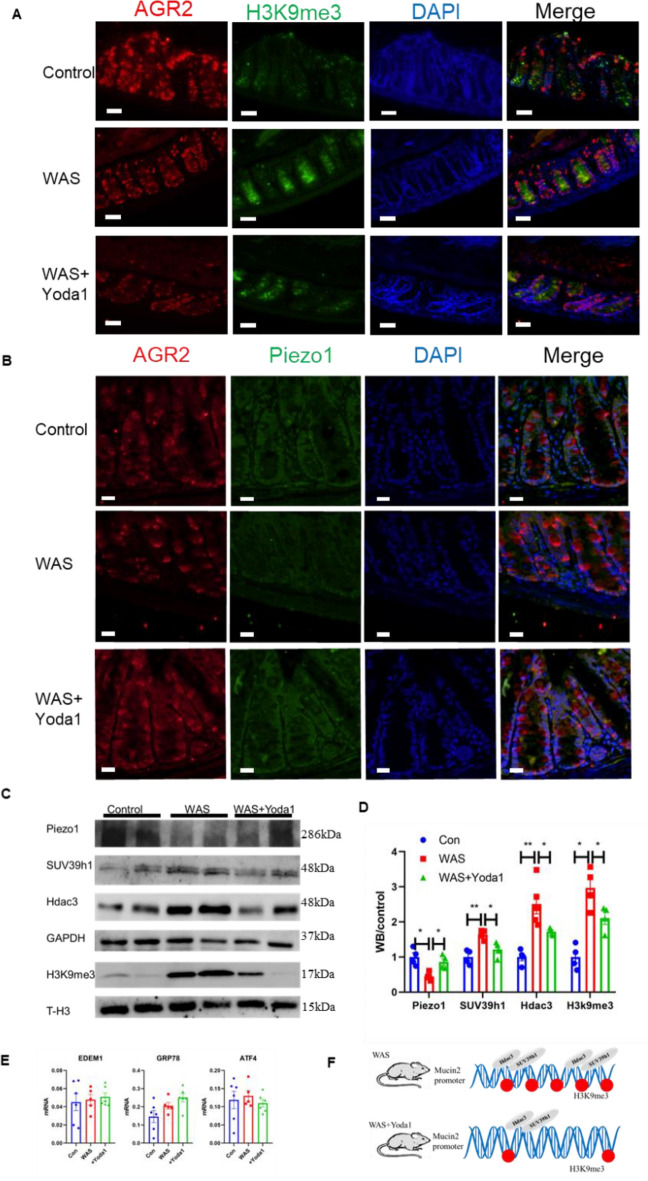
Piezo1 agonist Yoda1 reduced the methylation of intestinal mucosa in WAS model mice. **A** Immunofluorescence for co-localization of AGR2 and H3K9me3 in intestinal mucosa of control, WAS and WAS + Yoda1 mice; bar length: 20 μm. **B** Immunofluorescence for co-localization of AGR2 and Piezo1 in intestinal mucosa of control, WAS and WAS + Yoda1 mice; bar length: 20 μm. **C** Western blot of Piezo1, Suv39h1, Hdac3, GAPDH, H3K9me3 and T-H3 in the control and WAS mice (n ≥ 6). **D** Statistical analysis of protein in panel **C**. **E** RT-PCR of Piezo1, EDEM1, GRP78, ATF4 mRNA in the mucosa of control, WAS and WAS + Yoda1 mice (n ≥ 6). **F** Schematic showed that Yoda1 reduced the H3K9me3 modification on the mucin2 promoter by reducing Hdac3 and SUV39h1

We further detected changes in methylation in the mice exposed to WAS after Yoda1 intervention. Immunofluorescence showed that H3K9me3 methylation in goblet cells decreased after Yoda1 intervention, while Piezo1 expression increased slightly (Fig. 5A, B). WB confirmed that SUV39h1 and HDAC3 both decreased (Fig. 5C, D) and Piezo1 protein expression increased slightly in the Yoda1 group (Fig. 5C, D). In addition, we did not find significant changes in endoplasmic reticulum stress-related signaling molecules, including EDEM1, GRP78, and ATF4, in the WAS group or Yoda1 group compared to the control group (Fig. 5E), thus excluding the effect of endoplasmic reticulum stress on mucus expression.

### Decreased mucus expression and increased methylation in the Piezo1 flox-mucin2 Cre mice

**Fig. 6:**
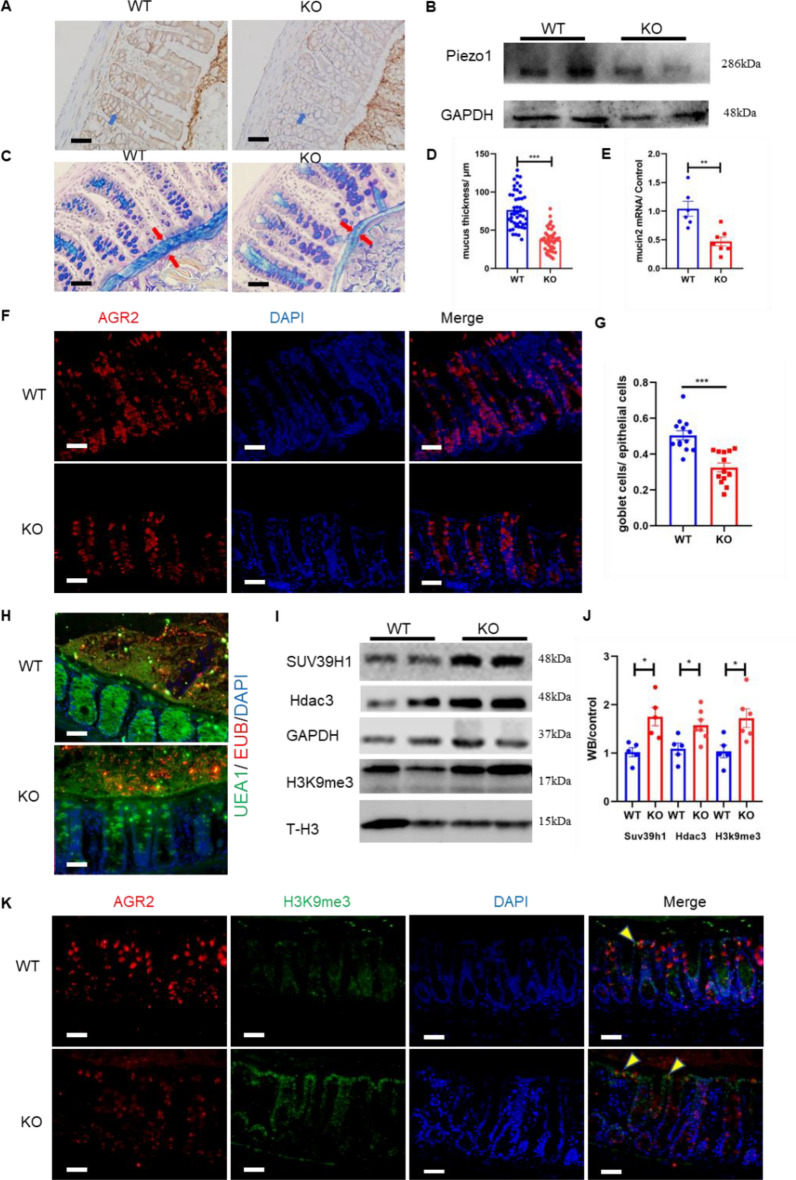
Decreased mucus expression and increased methylation in the Piezo1 flox-mucin2 Cre mice. **A** Immunohistochemistry of Piezo1 in WT and Piezo1 flox-mucin2 Cre mice (KO mice); bar length: 20 μm. **B** Western blot of Piezo1 in WT and KO mice. **C**, **D** Alcian staining and statistical analysis of mucus thickness in WT and KO mice (n ≥ 6); bar length: 50 μm. **E** RT-PCR of mucin2 in WT and KO mice (n ≥ 6). **F**, **G** Immunofluorescence and statistical analysis of goblet cells/ epithelial cells in WT and KO mice (n ≥ 6); bar length: 20 μm. **H** FISH of WT and KO mice, mucus was stained with UEA1 (green), bacteria were detected by fluorescence in situ hybridization with general bacterial 16S probes EUB (red) and DNA stained using DAPI (blue); bar length: 20 μm. **I**–**J** Western blot and statistical analysis of SUV39h1, Hdac3 and H3K9me3 in the colon mucosa of WT and KO mice. **K** Immunofluorescence for co-localization of AGR2 (label of goblet cells) and Piezo1 in intestinal mucosa of WT and KO mice; bar length: 20 μm

Next, we generated Piezo1 flox-mucin2 Cre mice (KO mice) to specifically knock out Piezo1 in colonic goblet cells. The success of Piezo1 knockdown in the KO mice was verified by immunohistochemistry and Western blotting (Fig. 6A, B). The thickness of the mucus layer decreased significantly, and mucin2 mRNA expression decreased slightly in the KO mice (Fig. 6C–E). We also found that the number of goblet cells decreased slightly after Piezo1 knockout (Fig. 6F, G). The results of FISH further confirmed the impaired mucus barrier function of the KO mice (Fig. 6 H). The methylation related protein SUV39h1, Hdac3 in mucosal tissue of model mice was significantly higher than that of wild type mice. In addition, we isolated mouse colonic mucosa for WB test of H3K9me3. Considering that there were kinds of intestinal epithelial cells in mucosal tissue, we further used goblet cell specific antibody AGR2 and H3k9me3 for fluorescence co-localization experiment. The results of WB and IF collectively confirmed that the expression of H3K9me3 in goblet cells up-regulated, which was consistent with the trend of mucus change (Fig. 6I–K). We also found that the colonic mucus thickness and permeability of the KO mice remained unchanged before and after WAS stimulation (Additional file 1: Figs. S1, S2).

### Mechanical stimulation reduced methylation in LS174T cells

**Fig. 7:**
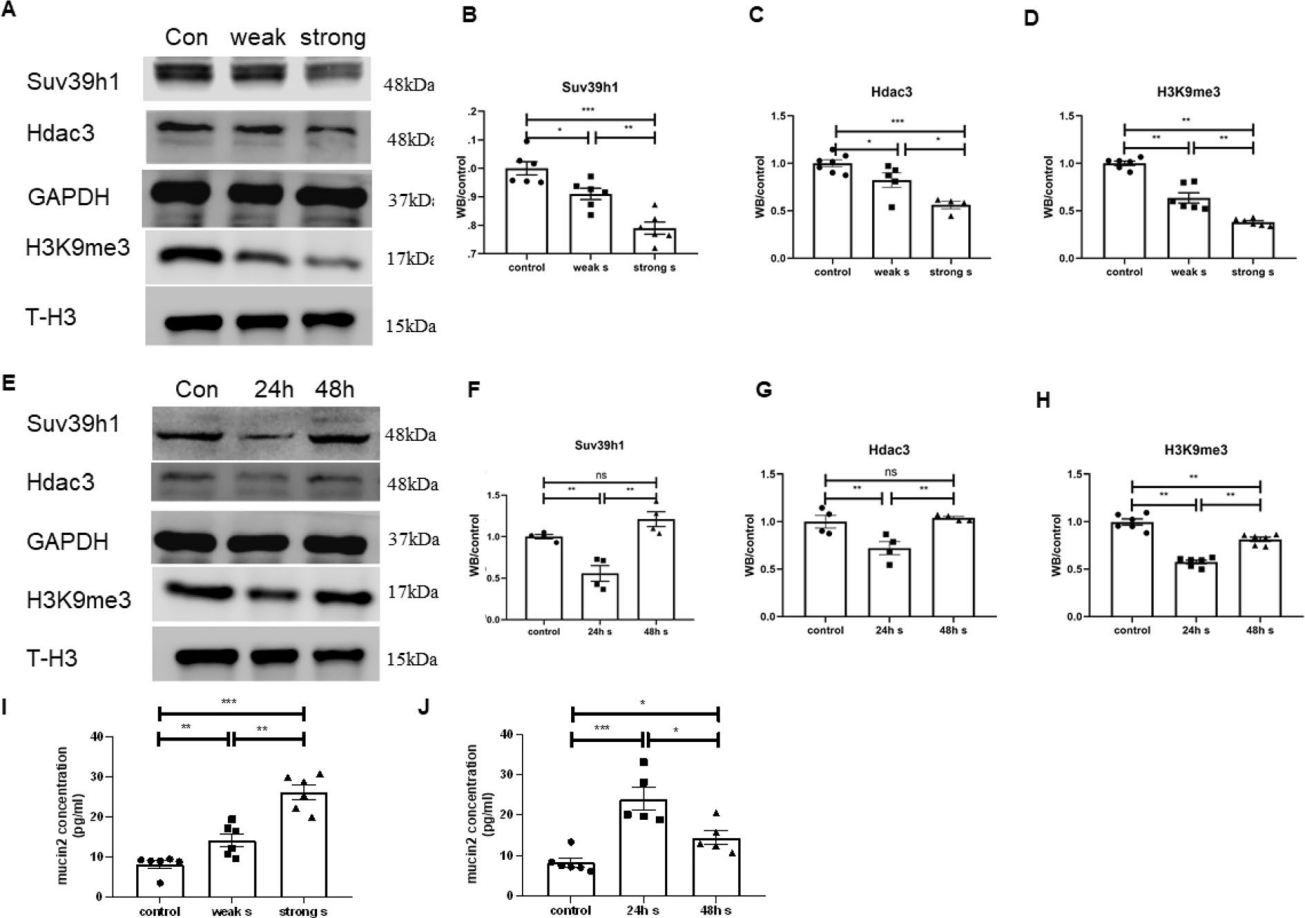
Mechanical stimulation reduced methylation in LS174T cells. **A**–**D** Western blots and statistical analysis of Suv39h1, Hdac3, GAPDH, H3K9me3 and T-H3 in LS174T cells before and after mechanical stimulation in different strength (n ≥ 4). **E** ELISAs of mucin2 in LS174T cells before and after mechanical stimulation of 24 and 48 h (n ≥ 5)

After verifying the relationship between Piezo1 and goblet cell methylation in the mice exposed to WAS, we cultured LS174T cells in a shaker board to simulate the effects of mechanical stimulation on goblet cells in the intestine. Mechanical stimulation activates Piezo1 to promote mucin2 expression in goblet cells has been proved [2]. Therefore, this study focused on the role of histone methylation in this process. We first administered different intensities of mechanical stimulation, and the results showed that the intensity of mechanical stimulation was negatively correlated with the level of methylation. The stronger the intensity of mechanical stimulation was, the more obvious the decrease in SUV39H1, Hdac3, and H3K9me3 was (Fig. 7A–D). Then, we maintained the intensity of mechanical stimulation at a strong level and changed the intervention time. The results showed that the levels of SUV39H1, HDAC3 and H3K9me3 decreased significantly after 24 h of intervention, and slightly recovered after 48 h of intervention, but still lower than the level before intervention (Fig. 7E–H). ELISAs showed that mechanical stimulation not only reduced methylation but also promoted the expression of mucin2, and there was a negative correlation between mucin2 and H3K9me3 (Fig. 7I, J).

### Inhibition of methylation by mechanical stimulation mediated by Piezo1

**Fig. 8:**
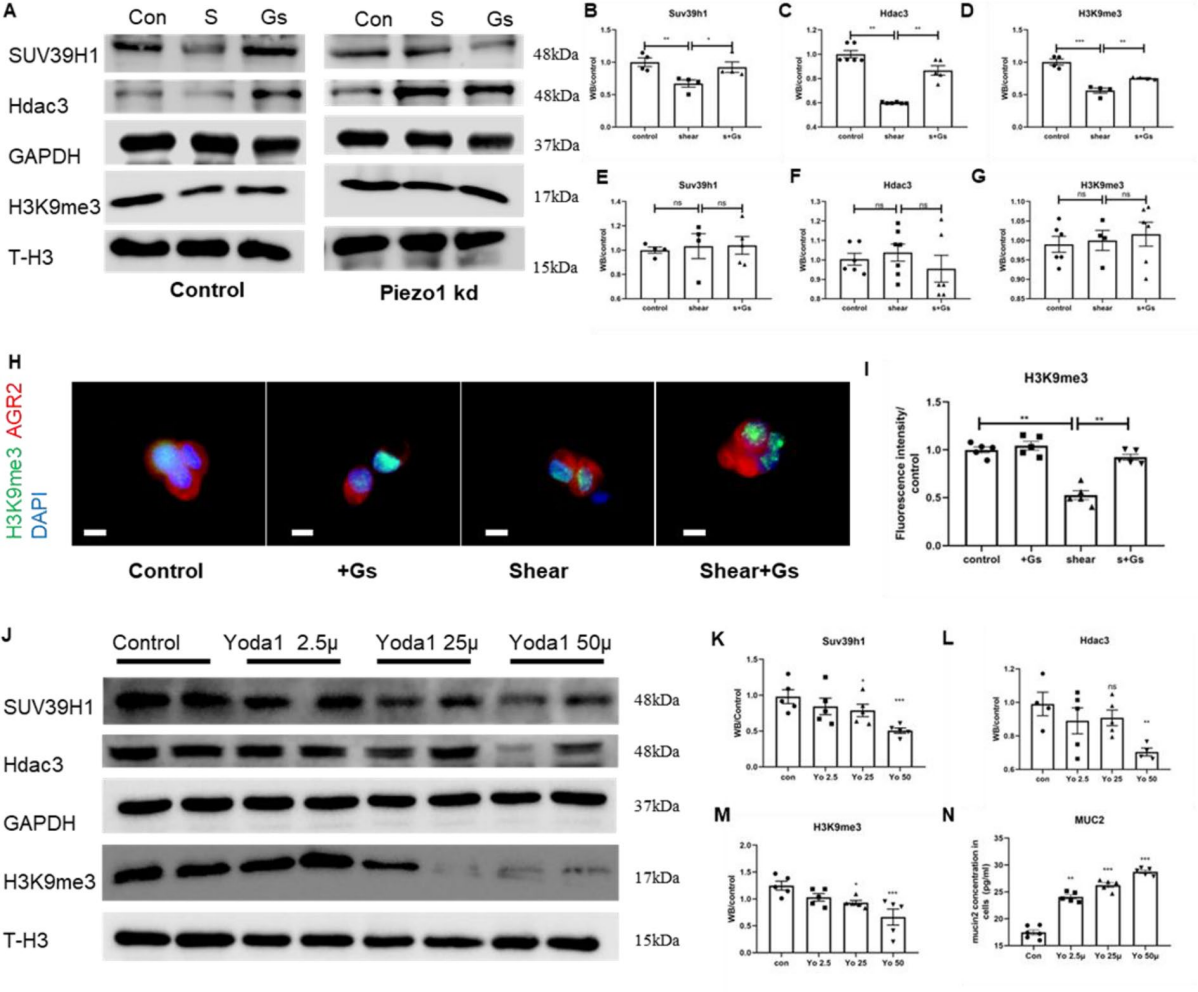
Inhibition of methylation of mechanical stimulation mediated by Piezo1. **A** Western blot of Suv39h1, Hdac3, GAPDH, H3K9me3 and T-H3 in in control and Piezo1 knock down LS174T cells before and after mechanical stimulation (n ≥ 6). **B**–**D** Statistical analysis of Suv39h1, Hdac3 and H3K9me3 protein in the control group in panel **A**. **E**–**G** Statistical analysis of Suv39h1, Hdac3 and H3K9me3 protein in the Piezo1-kd group in panel **A**. **H** Immunofluorescence for co-localization of H3K9me3 and AGR2 (label of goblet cells) in LS174T cells before and after mechanical stimulation; bar length: 20 μm. **I** Statistical analysis of H3K9me3 intensity in panel (H) (n ≥ 4). **J**–**M** Western blot and statistical analysis of Suv39h1, Hdac3, GAPDH, H3K9me3 and T-H3 in in LS174T cells before and after Yoda1 intervention (n ≥ 5). **N** ELISA analysis of mucin2 in LS174T cells after Yoda1 intervention (n ≥ 6)

Whether Piezo1 on the LS174T cells (Additional file 1: Fig. S3) is a key mechanical transduction signal molecule in the process of mechanical stimulation to inhibit methylation remains unclear. To solve this problem, we added the Piezo1 inhibitor GsMTx4 during mechanical stimulation, and the GsMTx4 intervention was sufficient to inhibit the expression of mucin2 under mechanical stimulation. WB detection showed that after intervention with GsMTx4, the inhibition of methylation by mechanical stimulation was significantly blocked (Fig. 8A–D). The immunofluorescence results were consistent with those of WB (Fig. 8H, I).

Considering that GsMTx4 does not strictly specifically act on Piezo1, we constructed specific Piezo1-kd LS174T cells by lentivirus transfection. The results showed that the inhibitory effect of mechanical stimulation on methylation was blocked after Piezo1 knockdown (Fig. 8A, E–G). We further treated LS174T cells with the Piezo1 agonist Yoda1. The results showed that Yoda1 downregulated the expression of Hdac3, SUV39h1 and H3K9me3 in a concentration-dependent manner (Fig. 8J–M), accompanied by a significant increase in the concentration of mucin2 protein (Fig. 8N).

### Mechanical stimulation reduces the binding of H3K9me3 to mucin2 promoter through Piezo1

**Fig. 9:**
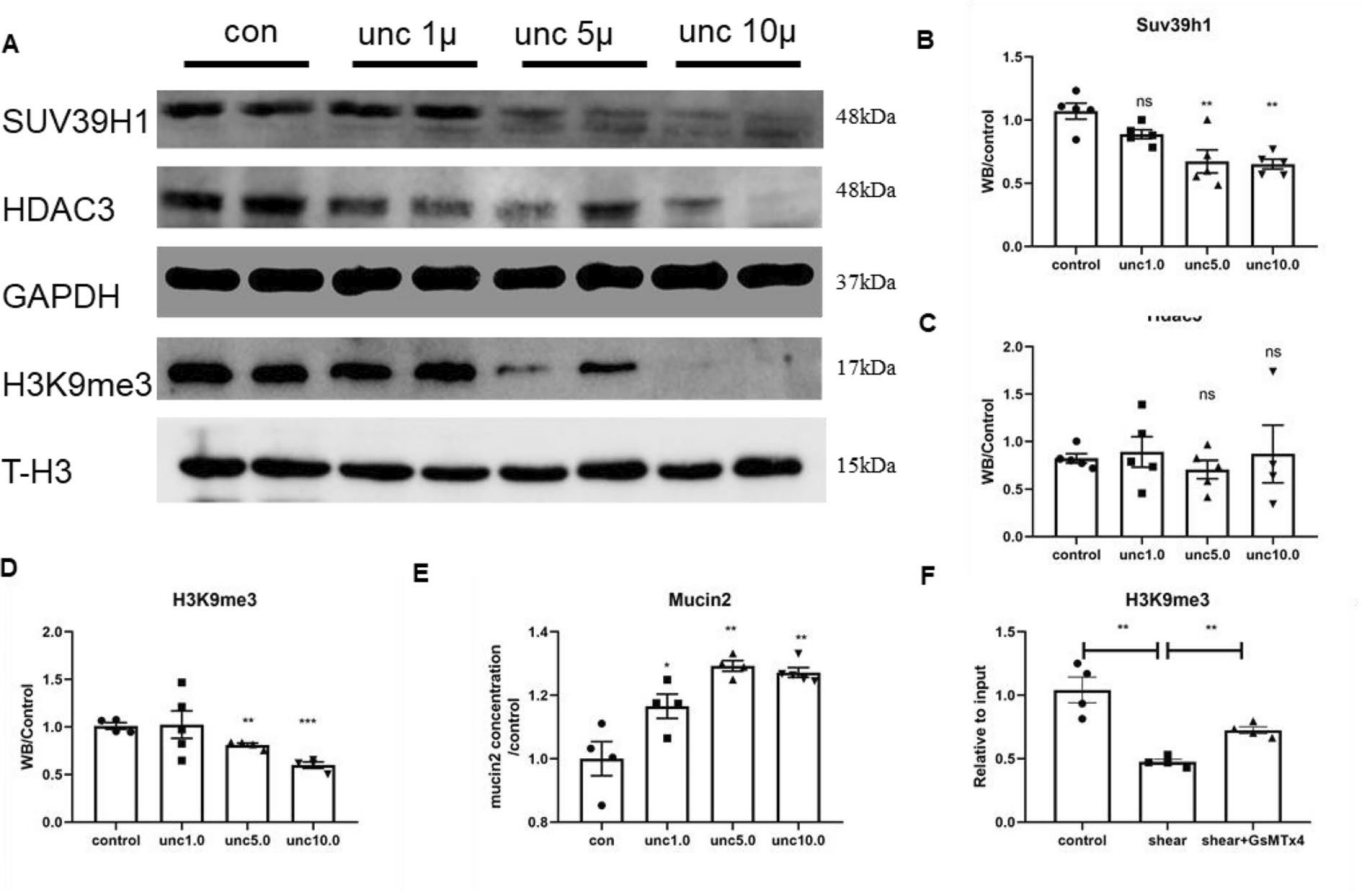
Mechanical stimulation reduced the binding of H3K9me3 to mucin2 promoter through Piezo1. **A**–**D** Western blot and statistical analysis of Suv39h1, Hdac3, GAPDH, H3K9me3 and T-H3 in LS174T cells before and after UNC0638 intervention (n ≥ 5). **E** ELISA of mucin2 in LS174T cells after UNC0638 intervention (n ≥ 4). **F** ChIP-PCR test of H3k9me3 on the mucin2 promoter in LS174T cells under shear and shear stress plus 0.1 μm GsMTx4 (n ≥ 4)

To verify the effect of H3K9me3 on the expression of mucin2 in LS174T cells, we added the H3K9me3 inhibitor UNC0638. In the absence of mechanical stimulation, UNC0638 inhibited the expression of SUV39h1 and significantly downregulated H3K9me3 levels in a concentration-dependent manner (Fig. 9A–D). The corresponding ELISA results showed that the expression of mucin2 in LS174T cells increased significantly after UNC0638 intervention (Fig. 9E).

To further confirm the mechanism of methylation of Mucin2 in LS174T cells, we detected the H3K9me3 on the mucin2 promoter under mechanical stimulation by ChIP-PCR. The results showed that compared with the basic conditions, strong mechanical stimulation lasting for 24 h could significantly reduce the level of H3K9me3 on the mucin2 promoter, while the addition of the Piezo1 inhibitor GsMTx4 to mechanical stimulation could alleviate this modification (Fig. 9F).

## Discussion

**Fig. 10:**
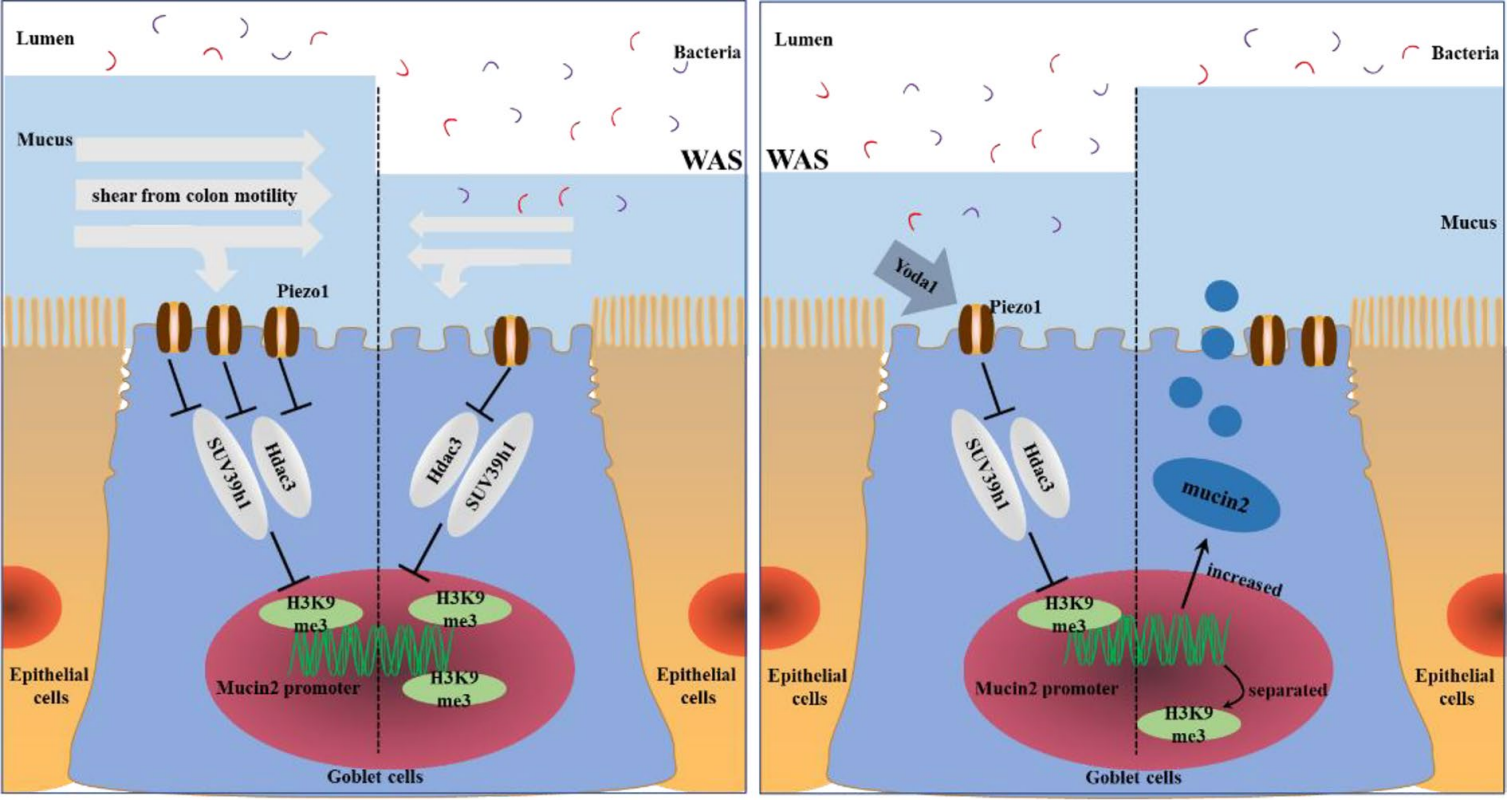
The schematic diagram of the mechanism by which the intestinal motility of WAS mice changes the colonic mucus barrier by acting on Piezo1 to regulate downstream methylation. Left side: Piezo1 on goblet cells of WAS mice decreased, and the decline of intestinal motility in WAS mice led to the downregulated signal of Piezo1 reception, which together led to the weakening of the inhibitory effect of piezo1 on downstream SUV39h1. Enhancement of SUV39h1 promoted H3K9me3 modification on the mucin2 promoter of goblet cells, resulting in the decrease of mucin2, mucus thickness and the increase in bacterial permeability. Right side: Addition of Yoda1 activates Piezo1 protein on intestinal goblet cells, inhibits downstream SUV39h1 and Hdac3, and reduces the binding of H3K9me3 to mucin2 promoter. After H3K9me3 isolation from mucin2 promoter, mucin2 gene was activated. Intestinal goblet cells synthesize and secrete more mucin2. The mucus thickness of WAS mice increased and the mucus barrier permeability improved

In this study, a series of findings confirmed that mechanical force reduced H3K9me3 modification in goblet cells and thus promoted intestinal mucus expression. In the mice exposed to WAS, the expression of Piezo1 in colonic mucosa decreased, accompanied by a decline in colonic contractile amplitude and peristaltic ability, together the mechanical stimulation signal sensed by Piezo1 on the colonic goblet cells decreased significantly. The weakened signal led to enhanced SUV39h1 expression and downstream H3K9me3 modification, which inhibited mucin2 gene transcription, resulting in defective colonic mucus expression. While addition of Yoda1 could activate Piezo1 on goblet cells to inhibit the expression of SUV39h1 and Hdac3 and downregulate the modification of H3K9me3, thus reducing the H3K9me3 level at the mucin2 promoter, activating the mucin2 gene and alleviating mucus barrier damage, improving mucus thickness and permeability (Fig. 10). We also confirmed that the colonic mucus thickness and permeability of Piezo-1 KO mice remained unchanged before and after WAS stimulation. Thus, we believe that this finding links abnormal intestinal motility and methylation in goblet cells in the mouse model of WAS through Piezo1, a mechanical transduction molecule, and enhances the understanding of intestinal pathological mechanisms in WAS and related IBS disease models.

Our study showed that the mucus thickness and mucus secretion rate of colonic mucus in WAS model mice were lower than those in the control group, accompanied by a decrease in mucin2 mRNA expression. In previous studies, intestinal mucus changes were rarely reported in IBS model mice, including mice exposed to WAS, and only a few studies indicated that the type of glycosylation modification of mucin changed in colonic mucus of IBS model mice [30]. In a rat model, the number of intestinal goblet cells and mucin2 gene expression decreased significantly when the xiphoid process was placed in water (1 h a day for 2 weeks) [29]. Differences in experimental results may be related to animal strains, but they may also be related to changes in intestinal flora and potential inflammation. Some scholars believe that one of the common points between IBS and IBD is damage to the mucosal barrier caused by changes in the intestinal flora [31, 32]. Compared to transepithelial resistance and fluorescence-labeled dextran assay, FISH test is more able to reflect early mucus changes [27]. We confirmed by FISH that the mucus barrier function of the WAS model mice decreased and the intestinal bacteria invaded into the mucus layer, FISH showed that the bacteria did not invade the intestinal crypts, it was likely to be the early stage of the destruction of the mucus barrier, which coincided with no obvious inflammation in the intestinal epithelium. We believe that this may be an early manifestation of intestinal inflammation in IBS reported in some studies [33].

Although it has been reported that intestinal contraction is usually enhanced in rat models of WAS [34], we found that colonic contraction in the mice exposed to WAS was decreased and insensitive to acetylcholine. The decrease in neural activity and depression in the mice exposed to WAS may be the underlying mechanism of this phenomenon [35, 36]. In addition, different animal strains may also cause some differences. Interestingly, we found colonic motility in the WAS mice model was positive correlated with mucus thickness, which indicated intestinal machinal signals influenced the mucus barrier function.

We believe that there is a positive correlation between intestinal motility and mucus secretion. In this study, we observed that the mucus thickness and mucus secretion rate of WAS mice were significantly lower than those of the control group, which was consistent with the trend of intestinal motility weakening. In our research, intestinal motility enhancement in WAS mice after the activation of Piezo1 was observed. It is not clear whether activating Piezo1 directly promotes intestinal motility, and Piezo1 is not expressed in smooth muscle cells. However, the piezo1 expressed in the intestinal ENS ganglion [37] may be activated by Yoda1 stimulation and further promote intestinal movement, which may be the potential mechanism of intestinal motility improvement in WAS mice after the addition of Piezo1 agonist, and we will explore it in our follow-up research.

Various forms of mechanical stimulation can regulate methylation modification, in which the specific mechanical transduction pathway is still the focus of attention [12, 13]. As a mechanically sensitive ion channel, Piezo1 can participate in mechanical signal transduction in a variety of tissues and cells [3], and some studies have shown that Piezo1 can upregulate histone deacetylase expression [38]. Our previous study has found piezo1 on goblet cells participate the synthesis and secretion of mucus [2], in this study, we further confirmed that mechanical force can alter histone methylation through Piezo1 protein in colon goblet cells. The effects of mechanical signals such as mechanical tension and shear stress on stem cell differentiation and cell reprogramming by regulating histone modification have been studied [39]. However, no study has pointed out whether mechanical force affects epigenetic involvement in the physiological process of intestinal mucus secretion, nor has it pointed out the role of Piezo1 protein. In our study, we found that the mechanical signals produced by intestinal peristalsis and contraction are positively correlated with mucus expression. More important, in the Piezo1 flox-mucin2 Cre model mice, the down-regulation of Piezo1 is accompanied by the decrease of mucus. Activating Piezo1 can improve intestinal motility and mucus barrier at the same time. Cell experiments also confirmed that activating Piezo1 inhibited two key enzymes in methylation modification, SUV39h1, Hdac3, and reduced the binding of H3k9me3 to the mucin2 promoter. Therefore, our results prove that Piezo1 links mechanical signals to epigenetic modification.

Although transient receptor potential cation channels (TRPs) and acid-sensing ion channels (ASICs) are also considered to be mechanically sensitive [40, 41], the expression of Piezo1 in intestinal goblet cells is higher than that of other mechanically related ion channels [2]. Piezo1, which directly senses changes in cell membrane tension and changes the molecular conformation of channels from the closed conformation to the open conformation, is a key mechanical transduction molecule in goblet cells. Notably, 24-h shear stress stimulation can more effectively inhibit methylation than 48 h, which may be the adaptive regulatory mechanism of the cell itself. As noted in the literature [42], when the cell is pulled, heterochromatin in the nucleus is secreted out of the nucleus in a short time to help the cell adapt to changes in the microenvironment and gradually recovers after the cell adapts.

Epigenetic studies of IBS focus on DNA methylation and histone modification. Previous studies have generally concluded that in the IBS model, especially in the WAS model, enhanced DNA methylation is closely related to visceral hypersensitivity, involving genes such as Par-3 family cell polarity regulator (Pard3), glucocorticoid receptor (Nr3c1), and cannabinoid receptor 1 (Cnr1) [28, 43]. Moreover, studies have shown that transient receptor potential cation channel subfamily V member-1 (TRPV1)-enhanced histone 3 acetylation in the spinal dorsal root ganglion is involved in the development of visceral hypersensitivity [28]. However, there are few studies on epigenetic changes in the intestinal barrier in a WAS model, and there are no reports on the mucosal barrier in WAS model mice. In the experiment, we selected methylation inhibitor UNC0638 to confirm that inhibition of methylation affects the expression of mucin2 in LS174T cells. The results combined with the inhibition of methylation by mechanical stimulation mediated by Piezo1 confirmed that the effect of activating Piezo1 is similar to that of UNC0638, both could inhibit H3K9me3. Besides, deacetylase Hdac3, methyltransferase SUV39h1 and methylated histone H3K9me3 increased synchronously under mechanical stimulation. Acetylation modification can be removed by Hdac3, and Suv39h1, the histone methyltransferase, contributes to methylation [44, 45]. Hdac3 deacetylation exposes the modification site, and SUV39h1 methylates it, which then affects the final H3K9me3 and completes the transformation process of silencing the activated gene.

Although the epigenetic modification form and transduction pathway of colonic goblet cells in WAS model mice may not be unique [3], our study combined cell experiments and animal experiments and used ChIP-PCR experiments to fully demonstrate the regulatory effect of Piezo1-SUV39h1-H3K9me3 on mucus expression. We have confirmed that mechanical stimulation could activate Piezo1 to promote mucin2 expression in goblet cells, based on the previous studies [2, 28], this research further showed that a close relationship between H3K9 methylation and mucin2 expression in goblet cells is mediated by Piezo1. We believe that this Piezo1-SUV39h1-H3K9me3 pathway plays an important role in the change of mucus during colonic motility dysfunction, which is expected to provide a new idea for the follow-up study of intestinal barrier impairment in IBS model, and improve the understanding of intestinal homeostasis regulation.

## Supplementary Information

**Additional file 1: Figure S1.** Alcian staining and mucus thickness statistics of Piezo1 flox-mucin2 Cre mice colon before and after WAS stress; bar length: 50μm.** Figure S2.** FISH of Piezo1 flox-mucin2 Cre mice colon before and after WAS stress. Mucus was stained with UEA1 (green), bacteria were detected by fluorescence in situ hybridization with general bacterial 16S probes EUB (red) and DNA stained using DAPI (blue); bar length: 50μm and 20μm.** Figure S3.** Immunofluorescence for co-localization of AGR2 and Piezo1 in LS174T cells; bar length: 20μm.

## Data Availability

All data generated or analysed during this study are included in this published article.
