# Supplementary material for: Activation of goblet cell Piezo1 alleviates mucus barrier damage in mice exposed to WAS by inhibiting H3K9me3 modification
Source: Cell Biosci. 2023 Jan 12;13:7. doi: 10.1186/s13578-023-00952-5 (PMC9835388; doi:10.1186/s13578-023-00952-5)
Supplement: Supplementary file 1 — Additional file 1: Figure S1. Alcian staining and mucus thickness statistics of Piezo1 flox-mucin2 Cre mice colon before and after WAS stress; bar length: 50μm. Figure S2. FISH of Piezo1 flox-mucin2 Cre mice colon before and after WAS stress. Mucus was stained with UEA1 (green), bacteria were detected by fluorescence in situ hybridization with general bacterial 16S probes EUB (red) and DNA stained using DAPI (blue); bar length: 50μm and 20μm. Figure S3. Immunofluorescence for co-localization of AGR2 and Piezo1 in LS174T cells; bar length: 20μm. [file 13578_2023_952_MOESM1_ESM.docx]

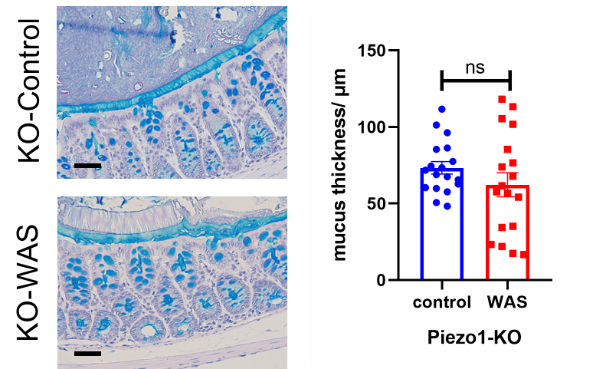


Additional figure 1. Alcian staining and mucus thickness statistics of Piezo1 flox-mucin2 Cre mice colon before and after WAS stress; bar length: 50μm.


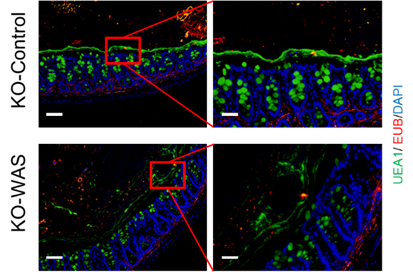


Additional figure 2. FISH of Piezo1 flox-mucin2 Cre mice colon before and after WAS stress. Mucus was stained with UEA1 (green), bacteria were detected by fluorescence in situ hybridization with general bacterial 16S probes EUB (red) and DNA stained using DAPI (blue); bar length: 50μm and 20μm.


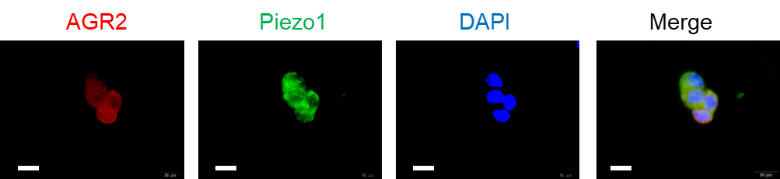


Additional figure 3. Immunofluorescence for co-localization of AGR2 and Piezo1 in LS174T cells; bar length: 20μm.
